# The Effect of Dietary Patterns and Nutrient Intake on Oxidative Stress Levels in Pregnant Women: A Systematic Review

**DOI:** 10.3390/antiox12071427

**Published:** 2023-07-15

**Authors:** Samar El Sherbiny, Giulia Squillacioti, Nicoletta Colombi, Federica Ghelli, Elena Lenta, Cloè Dalla Costa, Roberto Bono

**Affiliations:** 1 Department of Public Health and Pediatrics, University of Turin, 10126 Turin, Italy; samar.elsherbiny@unito.it (S.E.S.); federica.ghelli@unito.it (F.G.); roberto.bono@unito.it (R.B.); 2 Biblioteca Federata di Medicina Ferdinando Rossi, University of Turin, 10126 Turin, Italy; nicoletta.colombi@unito.it; 3 Clinical Nutrition Unit, Michele and Pietro Ferrero Hospital, 12060 Verduno, Italy; elenta@aslcn2.it (E.L.); cdallacosta@aslcn2.it (C.D.C.)

**Keywords:** oxidative stress, public health, pregnancy, dietary patterns, biomarkers, antioxidants, diet

## Abstract

During pregnancy, reactive oxygen species (ROS) may physiologically increase due to changes and growth of mother and fetal tissues. Consequently, oxidative stress (OS) may occur and be involved in the onset of pregnancy and newborn complications. Among exogenous antioxidant sources, diet is a cost-effective prevention strategy supporting the health of mothers and newborns; however, there is still a lack of nutritional education during pregnancy interviews. This review aims to systematically summarize the knowledge on the association between OS and diet during pregnancy. Four electronic databases (PubMed Central, EMBASE, Web of Science, and Food Science and Technology Abstracts) were searched on 22 December 2022. Among 4162 records, 13 original articles were finally included. Overall, 80% of the studies considered dietary patterns as exposure and 60% of them assessed the association with malondialdehyde levels in blood and urine. Three studies analyzed the influence of daily intakes of fruit and vegetables on different OS biomarkers (malondialdehyde, nitric oxide and 8-hydroxy-2′-deoxyguanosine). Among studies exploring dietary fat intakes (39%), 80% focused on polyunsaturated fatty acids, finding a positive association with glutathione peroxidase, biopirryn and isoprostane levels, respectively. Four studies analyzed vitamin intakes and 50% of them in association with 8-hydroxy-2′-deoxyguanosine.

## 1. Introduction

In 2016, the World Health Organization (WHO) provided global guidelines for a positive pregnancy experience. Among the 49 recommendations listed therein, 14 pertained to nutritional interventions. According to the WHO, maintaining a healthy lifestyle during pregnancy is crucial, and dietary counselling may support preventive strategies against potential pregnancy complications [1]. Conditions such as gestational diabetes mellitus, pre-term delivery [2], small-for-gestational-age babies [3], and pre-eclampsia [4], are associated with increased levels of oxidative stress (OS) during pregnancy. Although OS physiologically increases due to changes in maternal tissues and fetal growth [2,5], higher OS levels may be involved in several pathological conditions affecting both mothers and babies [5]. The adoption of a healthy and balanced diet is an affordable and natural way for humans to effectively increase the intake of large quantities of exogenous antioxidants [6]. Thus, effective primary prevention can also be achieved through a healthy and balanced diet, especially if rich in antioxidants. Dietary patterns are defined as the quantities, proportions, variety, and combination of different foods, as well as their consumption frequency [7,8]. Although traditional approaches used to investigate the impact of nutrition on health encompassed the analysis of a single or a few nutrients [9], nowadays the overall dietary model is applied to overcome some inherent limitations of the single-nutrient approach (e.g., the effect of a single nutrient can be too small to be detected) [8]. For this reason, the literature on this topic has increased in recent years and become fundamental for public health. Maternal nutrition during pregnancy is a major determinant of birth outcomes and, consequently, offspring health outcomes later in life. Moreover, diet can represent a useful, cost-effective and safe intervention to prevent most of the aforementioned OS-related conditions, during a time in which many pharmaceutical interventions are limited [10]. The association between maternal dietary patterns and infant birth outcomes has been summarized in previous reviews, with a specific focus on the relationship between dietary patterns and inflammatory state [11,12]. However, the relationship between maternal adherence to specific dietary patterns, maternal macronutrient intake and OS levels during pregnancy has not been evaluated yet [13]. Therefore, the aim of this work is to sum up the existing knowledge to fill the literature gap herein identified, and to verify the extent of the impact that diet can have on OS during pregnancy. Diet is a fundamental actor in the development of the fetus, and nutritional education should be introduced in the routine talk during pregnancy interviews.

## 2. Materials and Methods

This review was conducted according to the recommendations from Preferred Reporting Items for Systematic Reviews and Meta-Analyses PRISMA [14] and based on the registered PROSPERO protocol (Protocol n. CRD42023387270). Due to the prioritization of COVID-19 protocol registrations, the current submission passed a basic automated check and was published automatically.

### 2.1. Search Strategy

The search was conducted considering only original research. A preliminary search in PubMed, Embase and Web of Science supported the definition of specific keywords and gold-standard articles to be included in the review. Clinical trial registries, such as The World Health Organization International Clinical Trials Registry Platform (WHO-ICTRP), Clinicaltrial.gov (accessed on 14 December 2022) and the International Standard Randomized Controlled Trial Numbers (ISRCTN) registry, were also considered in this phase. This preliminary research enabled us to exclude randomized clinical trials and focus solely on observational studies due to a lack of suitable results for our work. In fact, the identified results were a small number and only single food were administered (e.g., yogurt or salmon), which was not in line with our goal. Additionally, we chose to avoid combined intervention with supplementation, as they can influence the biological results [15]. The search strategy was specifically tailored to each electronic database and was conducted on 22 December 2022. Four databases, PubMed Central, Embase, Web of Science and Food Sciences and Technology Abstracts (FSTA) were searched. The full search string is available in Appendix A. 

### 2.2. Eligibility Criteria 

The inclusion criteria for the studies were as follows: (1) original observational research; (2) involving pregnant women at any stage of gestation, (≥16 years); (3) measuring OS biomarkers in urine and/or blood; (4) including diet and/or nutritional habits as the main intervention/exposure; (5) published in English with a full text available. Systematic reviews, scoping reviews, expert opinions, editorials, conference abstracts and primary research reporting non-quantitative data, based on animal or in vitro experiments, were excluded. Additionally, studies involving any kind of antioxidant supplementation, pathological condition (e.g., gestational diabetes, pre-eclampsia, and hypertension,) or combined intervention (e.g., diet and physical exercise) including multiple pregnancies were excluded. Two independent reviewers carried out the screening process of titles, abstracts and full texts. 

### 2.3. Data Extraction 

Data were extracted and recorded in a customized Excel spreadsheet. The extracted information includes study design data (e.g., study type, study duration, aim, country, and main findings), population and sample information (e.g., sample size, age, Body Mass Index (BMI), and gestational age), details on diet (dietary pattern, nutritional habits, specific nutrients, and dietary assessment tool), and information about OS/antioxidant biomarkers (including units of measure, collection time, analytical method, and biological matrix). A particular focus was placed on extracting data regarding the association between diet and OS biomarkers. If different time points during the pregnancy were reported in the original paper, all the time points were extracted. Similarly, if different OS biomarkers or measurements at different time points were available, all of them were extracted. After the extraction process, a second reviewer checked data and all eventual discrepancies were discussed.

### 2.4. Quality Assessment 

The quality of the included articles was assessed based on the study design using appropriate checklists to evaluate the risk of bias (RoB) of the included articles. Specifically, the employed tools were: the National Institutes of Health (NIH) Quality Assessment Tool [16], for observational cohort, cross-sectional and case–control studies, and the NUtrition QUality Evaluation Strengthening Tools (NUQUEST) [17], a specific tool for evaluating RoB in nutritional studies. Two reviewers independently conducted the quality assessment and any discrepancies were discussed. Since the tools use different ratings, we expressed our evaluation as a percentage, and the final score was recoded based on tertiles (1st tertile 0–33% = poor quality; 2nd tertile 34–66% = medium quality; 3rd tertile 67–100% = high quality).

## 3. Results

**Figure 1:**
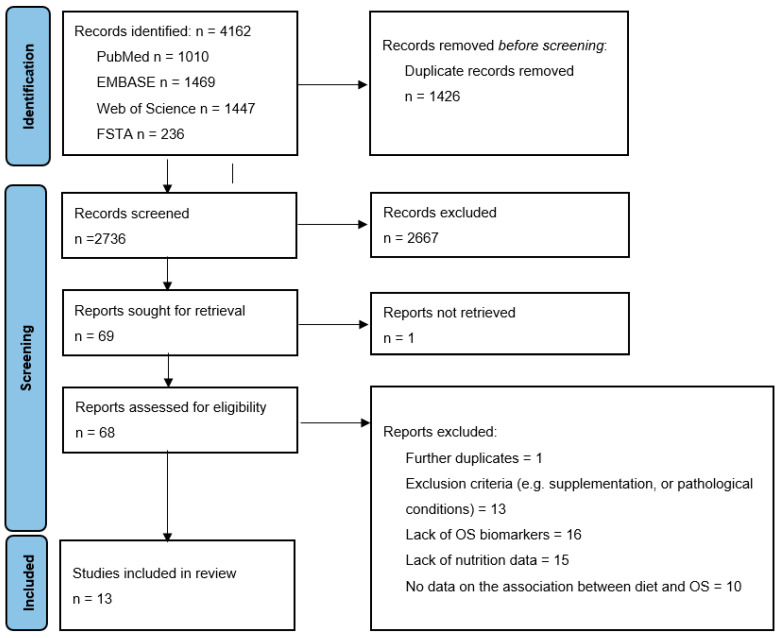
PRISMA flow diagram (Modified from: [15]). Abbreviations: FSTA: Food Science and Technology Abstracts; OS: oxidative stress.

A total of 4162 studies were initially identified from the databases used. After removing 1426 duplicates, the titles and abstracts of the remaining 2736 articles were screened based on the inclusion and exclusion criteria defined in the PROSPERO protocol. After the screening phase, 2667 studies that did not meet the inclusion criteria were excluded. At the end of the selection process, 69 papers were examined, and of these, 13 were included in this review. Figure 1 presents the entire process. The main reasons for exclusion relied on the absence of OS biomarkers and/or nutrition data, while other studies did not meet other eligibility criteria (e.g., age range, antioxidant supplementation, full text not available in English, studies conducted only on subjects suffering from a diagnosed disease).

### 3.1. Study and Participant Characteristics

Table 1 summarizes the characteristics of the studies. They were mainly conducted in the USA (n = 4), Mexico (n = 4), and Korea (n = 3). Only one study was located in Europe (Spain) and one in Japan, for a total of three continents and four countries. The publication dates range from 2001 to 2022. The majority of the studies used a cohort study design (69%) [13,18,19,20,21,22,23,24,25], while 23% employed a cross-sectional design [26,27,28], and only one study had a case–control design [4]. The study sample sizes ranged from 33 to 1158, totaling 5088 healthy women included in this systematic review, aged between 19 and 40 years. Only five articles (38%) reported the detailed educational distribution of the participants [13,18,19,22,24]. Among them, 84% achieved a middle/high education level. Participants’ BMI ranged between 18 and 35, and they were enrolled between 0 and 39 weeks of gestation. Two studies were omitted to report the gestational age of the participants at enrolment [23,28].

### 3.2. Biomarkers of OS

The main characteristics of the OS biomarkers and biological matrix are summarized in Table 2.

Overall, 46% of the studies measured OS biomarkers in spot urine, a non-invasively collected matrix, while 38% used blood. Only two studies (15%) used both blood and urine. In all the studies, a spot of urine was collected [4,13,18,20,22,24,25,26]; and in seven articles, a blood sample was collected. Among these, 57% used plasma, 29% used serum and in one study, whole blood was collected. A variety of analytical techniques has been used for the detection of OS biomarkers: Enzyme-linked immunosorbent assay (ELISA) (46%), colorimetric assays (31%), and High-Performance Liquid Chromatography (HPLC) (31%). Other techniques include Mass Spectrometry, CUPric Reducing Antioxidant Capacity (CUPRAC) and dinitropenylhydrazine (DNPH) (respectively [19,22]). The two most investigated biomarkers in relation to diet were malondialdehyde (MDA) (46%) and the DNA oxidation product, namely 8-hydroxy-2-deoxyguanosine (8OHdG) (31%). Considering the biological matrices, the most investigated OS markers in urine were MDA [18,24,26] and isoprostane [13,20,22], followed by 8OHdG. Only one study [25] investigated Biopyrrin and Coenzyme Q10 (CoQ10). In plasma, MDA and total antioxidant capacity (TAC) were the two most analyzed biomarkers, while 8OHdG, protein carbonylation (PC), and nitric oxide (NO) were only analyzed in one study, respectively. In serum and whole blood, the analyzed biomarkers were 8OHdG, glutathione peroxidase (GPx), and TAC. Only 23% of the studies collected more than one biological sample at different time points to measure the OS levels.

### 3.3. Dietary Assessment

Three main tools were used for the assessment of dietary intake of food and nutrients. The most frequently used was the 24 h recall method (38%), followed by the food frequency questionnaire (FFQ) (31%). Two studies (15% [13]) utilized a semi-quantitative FFQ, and another two studies used other questionnaires ([25,26]). Almost all the researchers that used a FFQ or one of its derivatives (SFFQ: semi-quantitative food frequency questionnaire; BDHQ: Brief Diet History Questionnaire) explained in detail the number of items that compose the questionnaire, the diet type, and the reference period during which it was administered. Only Ballesteros-Guzmán et al. [28] did not mention the number of items, diet type, and reference period. The items number of the FFQ and similar questionnaires, ranged from 62 [25] to 138 [24]. As per the 24 h recall method, Kim et al. [18] used a single recall, while all the other studies utilized three recalls [4,19,20,21]. The majority of the questionnaires have been administered during each trimester [4,19,20,21,25], three studies took into consideration the second trimester [13,22,26], and two studies referred to the third trimester [23,25,27]. Three studies did not specify the reference period of the questionnaire [26,28]. Overall, only 38% of the studies included in the present review applied multiple dietary measurements over time. The questionnaires were used mainly to assess the Mexican diet (23%) [19], the Korean diet (15%) [18,24], and the Western diet (31%) [4,20,21,22].

### 3.4. Dietary Patterns and Dietary Exposure Assessment

Dietary exposure was assessed using a questionnaire in all the included studies. Five of them [13,19,22,24,26] were able to identify dietary patterns starting from the questionnaire, while the others [4,18,20,21,23,25,27,28] assessed the consumption of fruit and vegetable, fat, and vitamins, expressed as g/day or g/1000 kcal.

Among the five studies that calculated dietary patterns only one [24] used a posteriori approach, based on the reduced-rank regression (RRR) method, to establish and define the patterns, starting from the data collected with the SFFQ. Morales et al. [13] used a priori-defined dietary index, which includes relative Mediterranean Diet (rMED), alternative Mediterranean Diet (aMED), Dietary Approach to Stop Hypertension (DASH), Alternate Healthy Index (AHEI), and AHEI-2010. Finally, Kim et al. [26] evaluated the frequency of meat and vegetables consumption, while Rodriguez-Cano et al. [19] appraised the ultra-processed food (UPF) consumption starting from the data collected according to the NOVA definition [29]. Tylavsky et al. [22] decided to evaluate the antioxidant power of the diet in relation with the OS levels, using an a priori method composed of the HEI score employed to validate the efficacy of the oxidative balance score they wanted to test.

Three studies [18,26,27] analyzed the impact of fruit and vegetable consumption on OS levels during pregnancy. Among them, Kim et al. decided to analyze the weekly frequency of consumption of vegetables, while in [27] and [18] was taken into account the daily consumption of both fruits and vegetables.

Another type of dietary exposure considered from five studies [4,19,20,21,25] was the consumption of total fats and polyunsaturated fatty acids (PUFA). All the articles except for Matsuzaki et al. [25], took into consideration the total fat intake and the fatty acid composition. [25] analyzed only the correlation between PUFA and OS. In particular, 40% of the studies took into account the total fat composition, the amount of PUFA, saturated fatty acids (SFA), and ω-3 and ω-6 daily intake. The 80% estimate PUFA intake, of this percentage, 75% consider the total daily intake of ω-3 and ω-6. A single study [4] estimates only the SFA consumption in addition to total fats without considering PUFA quantity.

Among the thirteen included studies, four [4,20,23,28] also reported the influence of vitamin intake on OS biomarkers. All of them measured the daily intake of vitamins A, C, and E.

### 3.5. Dietary Patterns, Nutrient Intakes and OS Biomarkers

#### 3.5.1. OS and Dietary Patterns

Overall, 39% of the studies considered dietary patterns as exposure exploring the association between OS reduction and a healthy diet (i.e., rich in fruits, vegetables, legumes, cereals, fish and olive oil with low consumption of red meat and alcohol, comparable to a Mediterranean pattern) [13,19,22,24,26]. Among them, 60% evaluated MDA. One study identifies a significant reduction in MDA levels in the presence of a “healthy diet” (*p* = 0.001) [24]. Another study [19] found controversial result: a significant decrease in MDA was detected in women consuming a UPF-rich diet (β = −0.0052, −0.007, −0.003, *p* < 0.0001), and a decrease in TAC in relation to UPF consumption was observed (β = −0.0005, −0.001, −0.000, *p* = 0.002). Daily higher intakes of fruits and vegetables, fiber, grain, nuts, and legumes were associated with significant lower levels of MDA (Spearman’s rho = −0.061, *p* < 0.01) [18], (Kruskal–Wallis test, *p* < 0.05) [27]. No significant associations were found between dietary patterns characterized by high consumption of meat and vegetables, and MDA levels [26]. The association between dietary patterns and 8OHdG was assessed by [13,26], but only [13] identified a significant result for the Mediterranean diet (β = −8.02, CI −15.4, −0.64, *p* for trend = 0.026). Tylavsky et al. [22], identified a significant decrease in isoprostane levels as the antioxidant power of the diet increase (*p* for trend = 0.0003).

#### 3.5.2. OS and Fruit and Vegetable

Kim et al. [18], Kim et al. [26] and Lopez-Yañez Blanco et al. [27] investigated the impact of fruit and vegetable intake on OS levels. All of them explored the association with MDA and two of them [18,27] identified also an inverse correlation between a higher intake of fruits and vegetables and a decrease in MDA levels (r = −0.061, *p* < 0.01) [18] (*p* < 0.05) [27]. NO decreased, which was inversely correlated with high fruit and vegetables intake (*p* < 0.05) [27], while 8OHdG showed no significant association with frequent consumption of vegetables (*p* = 0.323) [26].

#### 3.5.3. OS and Dietary Fats

Total fat and PUFA consumption was explored by five [4,19,20,21,25] out of the thirteen included studies. Four [4,20,21,25] over five studies observed a significant association between fat and PUFA amount in the diet, and OS biomarkers. Both [20,21] found that high levels of total fat, PUFA, ω6, and ω3 in the maternal diet were associated with OS increase. More specifically, they registered an increase in isoprostane excretion (*p* for trend (total fat and ω6) <0.05, *p* for trend (PUFA and ω3) <0.001) and GPx activity (Total fat: β = 0.045 ± 0.012, *p* < 0.001, PUFA: β = 0.104 ± 0.04, *p* < 0.03, ω3: β = 0.820 ± 0.022, *p* < 0.03, and ω6: β = 0.106 ± 0.05, *p* < 0.03), respectively. Similarly, 8OHdG was positively correlated with SFA levels (Pearson’ r = 0.38, *p* = 0.007) [4], and Biopyrrin, was higher in response to high intake of PUFA (β = −0.44, *p* < 0.001) [25]. Noteworthy, 75% of the studies that found a significant association observed it during the third trimester of pregnancy. Rodriguez-Cano et al. [19] did not identify any significant association with the three biomarkers analyzed (MDA, TAC, and PC).

#### 3.5.4. OS and Vitamins

Lastly, vitamins intake from food was examined by four studies [4,20,23,28]. Only two of them identified an association with OS. [28] found that maternal MDA was positively associated with vitamin C intake (*p* < 0.05). Diaz-Garcia et al. [23] highlighted that higher intake of dietary vitamin A is beneficial and can significantly reduce 8OHdG levels (Spearman’ rho = −0.445, *p* < 0.001). This result was not confirmed by Scholl [4], who analyzed vitamins A, C, and E, and did not find any significant reduction in DNA damage. Another study by Scholl et al. in 2005 [20] investigated the association of dietary vitamins intake and isoprostane and TAC levels. Again, they did not find any significant relationship. Two studies [4,20] verified also the impact of β-carotene and did not find any relation with OS. Table 3 reports results on the association between OS and dietary exposure. The most analyzed biomarker was MDA (46% of the studies). Of them, 83% identified a significant association between diet and MDA levels. Isoprostane was measured in 23% of the studies, with 67% of significant results, while 8OHdG was quantified in 31% of the studies, with 75% of significant results. TAC was assessed in 23% of studies and resulted to be the least correlated with diet (33%). Four studies used other biomarkers, 75% of them showed a significant result.

### 3.6. Risk of Bias (RoB) Assessment

The overall scoring was quite homogenous among the studies. The quality assessment according to NIH tool ranked 62% (n = 8) of the studies as “medium quality”, 31% as “high quality” (n = 4), and 8% as “poor quality” (n = 1) [28]. The NUQUEST scale instead classified 62% of the studies as “medium quality” (n = 8), 31% as “high quality”, and just one study classified as “poor quality” [26]. The mean score is in Table 1. We averaged the scoring from different tools obtaining 69% medium-quality studies, meaning that they could be affected by a certain degree of RoB, and 31% high-quality studies. The main weaknesses identified by both tools were a lack of multiple biomarker measures, and none of the studies provides a sample size justification or a power description. Only three studies provided information on the participation rate. These issues have been highlighted by both evaluation scales.

## 4. Discussion

This systematic review offers a summary of the current knowledge about the influence of diet on OS biomarkers during pregnancy. Different dietary habits and patterns were explored including the Mediterranean diet and the Western diet but also single-food and single-nutrient intakes such as fruits, vegetables, fats and vitamins. We observed that the contribution of dietary patterns on OS has been measured by different OS biomarkers. The most frequently measured biomarker was MDA, whose levels were significantly lower in association with patterns rich in fruit and vegetables. 8OHdG showed an increase in the presence of a diet rich in saturated fats and a decrease in relation to a Mediterranean pattern, which seems to be protective against DNA damage from free radicals, data already present in the literature [30]. Higher urinary isoprostane levels were associated with a Western diet, while in women who followed an antioxidant diet, isoprostane excretion was significantly lower. Biopyrrin increased considerably in relation to PUFA consumption, especially during the third trimester. Concerning the TAC and the enzymes involved in the antioxidant response, they were associated with UFP and high-fat consumption, respectively. NO was associated with higher intakes of fruit and vegetables consumption. No significant results were observed for other biomarkers such as PC and CoQ10.

Although the analysis of the overall diet could provide a better understanding of the effect of diet on OS and health, many studies still focus on single-nutrient analysis. In recent years, nutritional epidemiology moved to dietary pattern analysis and tried to buck the trend, but there is still a lack of trials and observational studies regarding overall pattern effects [31]. It is possible that the absence of a unique and validated method of pattern assessment led authors to focus on the single-nutrient approach. We observed that the most frequently used method was a priori, based on index calculations created from dietary recommendations. This approach can be useful to reduce the bias determined by the subjective reporting of the FFQ and 24 h recalls but is limited by the current knowledge behind index construction [8]. Another option is the a posteriori approach, which is dependent on what the subject declared in the questionnaire. In fact, the construction of the statistical model strictly depends on dietary data obtained on the basis of eating behavior. Questionnaires such as FFQ or 24 h dietary recall are the most common tools used to collect dietary information from people, but they have some limitations; people tend to underestimate the amount and quality of the foods that they consume, and the report is subjective [32]. Therefore, also in a posteriori-derived method, results might be biased. An example is the result obtained by Rodriguez-Cano and colleagues [19] about MDA levels in relation to UPF consumption. Their controversial result can be attributed to this phenomenon, along with the effect modification played by BMI (i.e., women with higher BMI and higher exposure to UPF could be expected to exhibit higher levels of MDA.) [33,34]. The dietary assessment method can expose to methodological issues able to produce biased results. To date many more studies on the relationship between UPF and MDA are required to clarify this point. A possible strategy to limit some methodological problem is to assess dietary consumption at different time points during the observation [32]. Another option could be combining different dietary assessment methods and, possibly, integrating them with a biological measure, (e.g., nutritional biomarkers) [35]. Hwang et al. [24] applied this method, by using some nutritional biomarkers such as serum folate, iron and zinc as intermediate response variables to derive the patterns by applying the RRR method. They observed a relation between dietary pattern 1 (balanced and rich in fruits, vegetables, grains and legumes), nutritional biomarkers and OS biomarkers. A similar approach has been applied by Tylavsky et al. [22], who derived an oxidative balance score (OBS), calculated on the basis of dietary anti and pro-oxidant power.

Another fundamental aspect of the study design is the choice of the OS biomarkers and the timing of biological sample collection. The use of a single measurement may produce pitfalls, as biomarkers quantified only at the beginning of gestation, are not representative of the overall trend of the pregnancy. Conversely, sample collection during the third trimester without a baseline measure does not consider that OS during the third trimester is physiologically higher [2]. The lack of repeated biological measurement can constitute an important source of bias. Repeated measures are essential to reduce measurement errors and flatten the physiological OS fluctuations during pregnancy. Despite all these possible methodological problems, the quality assessment suggested that most of the studies have medium or low risk of bias. We used different RoB tools with the aim of partly overcoming a potential RoB underestimation. Although the NUQUEST scale is specifically created to evaluate nutritional studies, we observed almost no difference between the scoring derived from NIH and the NUQUEST. This can be attributed to the fact that some studies did not have the evaluation of diet with respect to OS as the main outcome of the study.

**Figure 2:**
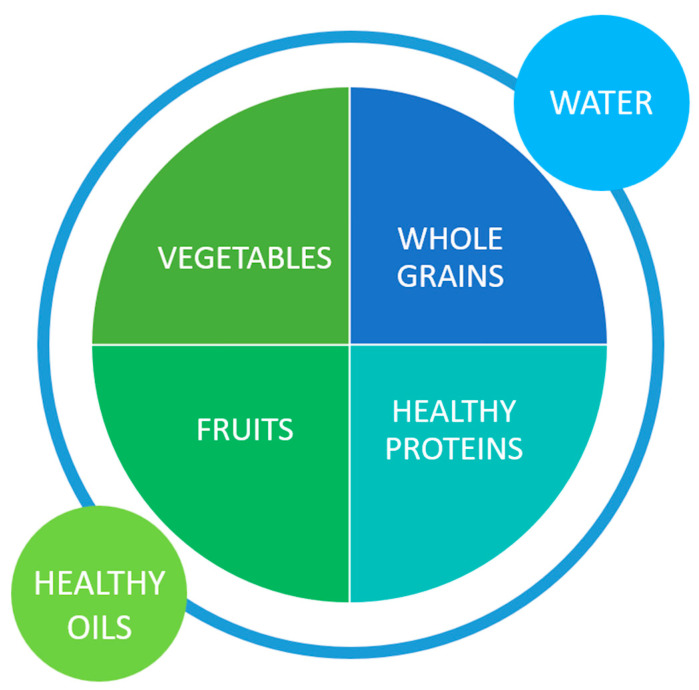
Healthy eating plate.

In addition to the analysis of overall patterns, the evaluation of fruit and vegetables consumption can be considered similarly important, since they are the main source of antioxidants (vitamins and non-nutrient sources) and fibers, and they are related to lower levels of OS [36]. The WHO recommendations for a healthy and balanced diet suggest to eat at least 400 g of fruit and vegetables per day, in order to assume all the essential micronutrients, and fibers [37,38]. Moreover, guidelines from Harvard University recommends a new way to make a healthy eating plate (Figure 2), in which fruit and vegetables represent half of the meal. Despite these recommendations, fruit and vegetables consumption during pregnancy is generally low [38,39]. Fruit and vegetables intake was assessed by three studies [18,26,27], and in two of them, a positive correlation was identified with the reduction in MDA. The only study that found no association with vegetables was [26], probably because it considered only the frequency of consumption per week, and fruit was not considered. Two groups [18,27] studied the impact of fruit and vegetable intake on MDA, in order to assess the possible relationship with OS. In both studies, a positive association was found, meaning that a significant reduction in MDA was detected in subjects who consume higher quantities of fruit and vegetables.

**Figure 3:**
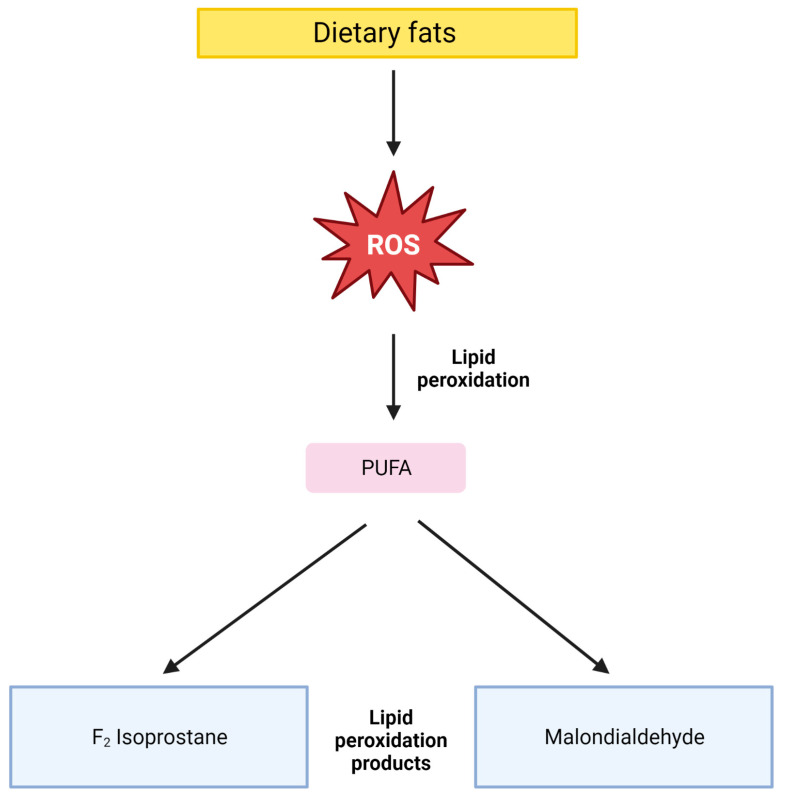
Suggested mechanism of dietary fats contribution to lipid peroxidation. Dietary fats may represent a source of substrates for lipid peroxidation products. ROS: reactive oxygen species; PUFA: polyunsaturated fatty acids (figure Created with BioRender.com).

With regard to dietary fat intake, three [20,21,25] out of four studies identified a significant association with OS during the third trimester. The increase in OS, due to fat catabolism, and the release of free fatty acids during the third trimester is in line with previous studies [2,40,41]. In addition, the influence of a diet rich in fats could contribute to this increase [42,43]. One of the main methods used to measure OS-derived damage in vivo is the assessment of lipid peroxidation products. PUFAs are susceptible to oxidative damage [44], so it is reasonable to expect that a high-fat diet, rich in PUFA, may contribute to OS increase by offering substrates for lipid peroxidation product creation such as MDA and isoprostane [45]. Hence, MDA and isoprostane can be useful OS biomarkers as suggested by the mechanism depicted in Figure 3. Although this phenomenon is theoretically plausible, many studies did not confirm our hypothesis [45,46]. Of the five studies that analyzed the association between dietary fats and OS, only two [19,20] quantify the lipid peroxidation products MDA and isoprostane, respectively. Isoprostane was positively associated with dietary fats intake [20], meaning that with the increase in total fat and PUFA consumption, an increase in urinary isoprostane was observed. Lipid peroxidation products can be used as OS biomarkers to assess the oxidative damage of a high-fat diet, but further studies are required, to clarify dietary fat role in their metabolism. TAC showed no significant correlation in both the studies that evaluated it [19,20] probably because both populations’ diets were characterized by UPF consumption or, broadly, we can assume they followed a Western diet model. The Western diet is typically associated with low consumption of high-quality and nourishing foods, such as non-refined carbohydrates (fruits and vegetables) and legumes proteins, essential for vitamins, minerals, nutrients, and antioxidants supply [43]. Therefore, the lack of antioxidants from the diet could have been balanced by the activation of enzymatic antioxidant defence. This latter hypothesis seems to be confirmed by Chen et al. [21], who quantified GPx and found a significant correlation between the increase in total fat, PUFA consumption, and increase in GPx activity during the third trimester. The last biomarker with a significant correlation with PUFA intake is byopirrin. Biopyrrin is an oxidized metabolite of bilirubin. Bilirubin is able to scavenge ROS, and the reaction products (biopyrrins) are excreted in urine [47]. Biopyrrin shows a significant increase with a high level of PUFA intake. The same study also suggested the possible influence of dietary PUFA on CoQ10. CoQ10 can be considered a marker of OS due to its strong antioxidant activity [25], but the association with diet was found not significant. Finally, Scholl et al. [4], analyzed the impact of SFA intake on DNA damage by using 8OHdG as a biomarker. The result is a significant correlation between the intake of SFA and DNA damage. In conclusion, from the included studies the most interesting OS biomarkers to evaluate the impact of high-fat diets are lipid peroxidation products, byopirrins, and GPx activity in relation to PUFA intake, while for the evaluation of the damage operated by SFA, 8OhdG seems to be effective. Further studies are required to clarify the impact of a high-fat diet on OS and to confirm the role of the aforementioned biomarkers.

**Figure 4:**
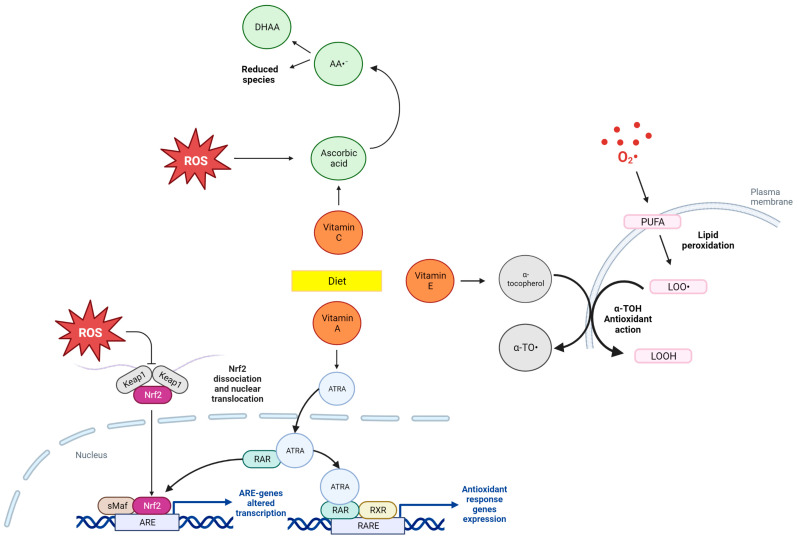
Dietary antioxidant mechanism of action of vitamin A, vitamin E and vitamin C (figure Created with BioRender.com). Abbreviations: ROS: reactive oxygen species; sMaf: small musculoaponeurotic fibrosarcoma proteins; ARE: antioxidant response element; ATRA: all-trans-retinoic acid; RAR: retinoic acid receptors; RXR: retinoid X receptor; RARE: retinoic acid response elements; α-TO∙: α-tocopheroxyl radical; α-TOH: α-tocopherol; LOOH: lipid hydroperoxide; LOO∙: lipid peroxyl radicals; PUFA: polyunsaturated fatty acids; AA: ascorbic acid; DHAA: dehydroascorbic acid.

Vitamins, and more specifically, vitamin C and E, are acknowledged for their antioxidant properties. Vitamin C is mostly present in fruit and vegetables and can exert its antioxidant role in aqueous environments. Despite all the evidence about their antioxidant actions, many trials report controversial information about their effectiveness in the reduction of OS [48]. More in depth, vitamin C has been reported as having a pro-oxidant effect that depends strongly on iron availability [49]. The pro-oxidant activity consists mainly in promoting DNA damage under pathological conditions. Despite this controversial function, vitamin C more often plays an antioxidant role [50] as proposed in Figure 4. Ballesteros-Guzmán et al. [28] found a positive correlation between vitamin C intake and MDA, confirming the pro-oxidant role of ascorbic acid. It is noteworthy that they did not give us some essential information about the dietary pattern that was taken into consideration, or about the timing of biological sample collection, so it is difficult to make any assumption about their results.

Vitamin A has an important antioxidant action too, but different from that of vitamins C and E. In particular, all-trans-retinol, which is the active metabolite of vitamin A acts as an indirect antioxidant, regulating the transcription of some genes involved in antioxidant response (e.g., NRF2 pathway) (Figure 4). Since this action is more complex than that of vitamins C and E which have a direct antioxidant activity (by quenching ROS, and preventing lipid peroxidation, respectively), this can explain why only one out of four studies identified a correlation between vitamin A intake and OS regulation. Another possible explanation of the lack of results could be that very often vitamin A is mistakenly considered a direct antioxidant vitamin, with a function similar to that of vitamins C and E. The only direct antioxidant activity of vitamin A, can be searched in provitamin A carotenoids such as β-carotene [51]. Both [4,20] quantified β-carotene, but did not find any significant relationship with OS biomarkers (isoprostane and TAC, and 8OHdG, respectively), as for vitamin A. [23] looks for a correlation between vitamin A and DNA damage operated by free radicals, through 8OhdG quantification, finding a significant association between them (A higher tertile of vitamin A consumption corresponds to a lower 8OhdG excretion). Given the all-trans-retinol regulatory function, we can expect to see a long-term effect compared to that of direct antioxidants. Thus, it would be useful to quantify the activity of enzymes such as GPx, SOD, and CAT in addition to the DNA damage.

Vitamin E (α-tocopherol) is an important antioxidant thanks to its ability in preventing lipid peroxidation, and as a ROS scavenger [51] (Figure 4). [4,20] evaluated vitamin E intake in an American population, characterized by the Western type of diet acknowledged for its pro-oxidant properties and high consumption of ultra-processed foods able to increase the OS and inflammatory state of the body. Hence, it is possible that the antioxidant effect of vitamins was covered by the pro-oxidant effect of the high fat consumption. In [23,28], the population was Mexican; from the literature, we know that the Mexican diet is similar to a Mediterranean pattern [52], but they failed in the identification of a correlation with OS too. Overall, the reason for the lack of significant results in vitamin influence on OS need to be searched in the fact that micronutrients effect could be too small to be detected. The positive results of Diaz-Garcia [23] can be due also to the fact that the global consumption of fruit and vegetables in their populations is higher than the American one.

From a broader point of view, we also need to keep in mind that people do not eat isolated nutrients, but all the nutrients assumed through diet can interact and can have a collaborative function inside our organism. The low percentage of significant results regarding OS and vitamins is more likely to be attributed to the overall effect that multiple nutrients of the meal have on the oxidative balance, rather than an actual lack of effectiveness of vitamins as antioxidants.

### Strength and Limitations

One of the main strengths is that the present systematic review followed a rigorous method. An a priori protocol has been established to ensure transparency and scientific rigor. Two reviewers carried out independently the screening phases, as well as the quality assessment of the studies. To the best of our knowledge, this is the first work that revises all the existing articles about the relationship of OS and diet during pregnancy. Another strength is the use of two different tools for the RoB assessment. By using the NUQUEST scale, we tried to overcome a critical issue, which is the lack of criteria to evaluate nutritional interventions in human nutrition studies. The main limitations of our study are the heterogeneity of the biomarkers and of the dietary patterns, which precluded us from conducting a meta-analysis. Moreover, the analysis of studies focused on single nutrients should be considered a limitation too, due to their aforementioned collaborative function inside our body.

## 5. Conclusions

Among many different biomarkers analyzed by the studies in this review, blood and urinary MDA seems to be the most influenced by both dietary patterns and micronutrients. As expected, a high intake of fruit and vegetables is protective from MDA increase, while a controversial result was found regarding diets rich in ultra-processed foods. Concerning other biomarkers, diets rich in fruit and vegetables seemed correlated with NO decrease. Some significant results have been obtained for isoprostane urine levels, which drops in the presence of an antioxidant diet and increases in the presence of the Western diet with high consumption of fats. DNA damage quantified by 8OHdG (in both blood and urine) shows an increase in correlation with a diet rich in saturated fats, and a decrease in relation to a Mediterranean pattern and vitamin A consumption. Another biomarker that shows a significant increase in relation to high fat consumption is biopyrrin. In terms of antioxidant capacity and antioxidant enzymatic response, only two studies gave positive results, respectively, in correlation to ultra-processed-food consumption and high fat ingestion. The remaining biomarkers, namely PC and CoQ10, show no correlation with diet. In conclusion, despite the heterogeneity of biomarkers analyzed in this review, diet is confirmed as an important factor in the modulation of OS during pregnancy. Further studies with an appropriate methodology and appropriate OS biomarker evaluation are required.

## Figures and Tables

**Table 1:** Study characteristics as reported by the original studies included in the systematic review. The risk of bias was firstly assessed using the National Institutes of Health (NIH) [16] Quality Assessment Tool and the NUtrition QUality Evaluation Strengthening Tools (NUQUEST) [17] checklist and then reported as average scoring among them.

Study Reference	Country	Study Design	Sample Size	Maternal Age (Mean ± s.d.)	Dietary Exposure/Assessment Method	OS Biomarkers	Main Findings	Risk of Bias
Ballesteros-Guzmán, A.K. 2019 [28]	Mexico	Cross-sectional	33	30.1 ± 3.6	Average macro and micronutrients/FFQ	MDA/TAC	No significant associations between maternal diet and MDA, TAC. ↑Vit C significantly associated with ↑MDA	Medium
Chen, X. 2003 [21]	USA	Cohort	408	29.6 ± 3.8	Dietary fat intake/24 h recall	GPx	↑PUFA, n-3 and n-6 FA significantly associated with ↑GPx activity	High
Diaz-Garcia, H. 2022 [23]	Mexico	Cohort	90	24.4 ± 5.5	Daily nutrients intake/FFQ	8OHdG	↑Vit A intake significantly related to ↓8OHdG levels	Medium
Hwang, J. 2021 [24]	Korea	Cohort	1158	32.7 ± 4.5	Dietary pattern 1, 2, 3/SFFQ	MDA	Dietary pattern 1 significantly associated to ↓MDA in urine	Medium
Kim, H. 2011 [18]	South Korea	Cohort	715	29.52 ± 4.97	Fruits and vegetables intake/SFFQ	MDA	Significant correlation between ↑fruit and vegetable intake and ↓MDA	Medium
Kim, Y. J. 2005 [26]	Korea	Cross-sectional	235	NA	Meat and vegetables consumption/Questionnaire	MDA/ 8OHdG	No significant differences between patterns and their association with OS	Medium
Lopez-Yañez Blanco A. 2022 [27]	Mexico	Cross-sectional	238	22.9 ± 0.78	Fruits and vegetables intake/FFQ	MDA/NO	↑ Fruit and vegetables intake significantly associated to ↓OS	Medium
Matsuzaki, M. 2014 [25]	Japan	Cohort	49	26.3 ± 5.4	PUFA intake/BDHQ	Biopyrrin/ CoQ10	↑ PUFA intake during the 3rd trimester significantly associated with ↑ biopyrrin	High
Morales, E. 2022 [13]	Spain	Cohort	665	20 to 40	Mediterranean diet, DASH diet, AHEI/FFQ	8OHdG/Isoprostane	↑ Adherence to rMED significantly associated with ↓8OHdG levels, ↑ adherence to DASH diet marginally associated to ↓ Isop	Medium
Rodriguez-Cano A. M. 2022 [19]	Mexico	Cohort	119	21.86 ± 0.23	Ultra-processed food consumption/24 h recall	TAC/MDA/PC	↓UPF intake significantly associated to ↑ TAC, UPF negatively associated with MDA	High
Scholl, T. O. 2001 [4]	USA	Case–control	52	24.1 ± 5.2	Dietary fat, vitamins intake/24 h recall	8OHdG	Significant correlation between ↑SFA and ↑8OHdG	High
Scholl, T. O. 2005 [20]	USA	Cohort	307	NA	Daily nutrients intake/24 h recall	Isoprostane/TAC	Maternal diet significantly associated with ↑Isop, ↑PUFA significantly associated with ↑Isop	Medium
Tylavsky, F. A. 2022 [22]	USA	Cohort	1019	31	OBS/FFQ	Isoprostane	↑ OBS significantly associated with ↓ Isop	Medium

↑ increase; ↓ decrease. Abbreviations: SFA: saturated fatty acids; PUFA: polyunsaturated fatty acids; FFQ: food frequency questionnaire; SFFQ: semi-quantitative food frequency questionnaire; BDHQ: Brief Diet History Questionnaire; OBS: oxidative balance score; MDA: malondialdehyde; NA: Not Available; TAC: total antioxidant capacity; PC: protein carbonylation; GPx: glutathione peroxidase; 8OHdG: 8-hydroxy-2′-deoxyguanosine; CoQ10: Coenzyme Q10; NO: nitric oxide, Vit: vitamin. Pattern 1: high consumption of grains, light-colored vegetables, legumes, fruits, meat, eggs, fish, and nuts. Pattern 2: low consumption of white rice, poultry, meat, and red meat by-products. Pattern 3: high consumption of grains, milk, and yogurt and low consumption of rice cake, legumes, snacks, bony fish, and tofu/soy milk. DASH: Dietary Approaches to Stop Hypertension; AHEI: Alternate Healthy Index; rMED: relative Mediterranean Diet; aMED: alternative Mediterranean Diet; UPF: ultra-processed food. Data on biomarkers with no significant associations with diet were not reported unless differently indicated.

**Table 2:** Oxidative stress biomarkers and biological matrices of the included studies.

Study Reference	Biological Matrix	OS Biomarker	Analytical Method
Kim, H. 2011 [18]	Urine *	MDA [µmol/g creatinine]	HPLC
Kim, Y. J. 2005 [26]		MDA [µmol/g creatinine]	HPLC
Kim, Y. J. 2005 [26]		8OHdG [µg/g creatinine]	ELISA
Scholl, T. O. 2001 [4]		8OHdG	ELISA
Tylavsky, F. A. 2022 [22]		Isoprostane	MS
Hwang, J. 2021 [24]		MDA [µmol/g creatinine]	HPLC
Matsuzaki, M. 2014 [25]		Biopyrrin [µmol/g creatinine]	ELISA
Matsuzaki, M. 2014 [25]		CoQ10 [ng/mL]	HPLC
Diaz-Garcia, H. 2022 [23]	Blood (Plasma)	8OHdG [ng/mL]	ELISA
Lopez-Yañez Blanco A. 2022 [27]		MDA [µmol/L]	Colorimetric (TBARS)
Lopez-Yañez Blanco A. 2022 [27]		NO [µmol/L]	Colorimetric (Griess assay)
Ballesteros-Guzmán, A.K. 2019 [28]		MDA [µmol/L]	Colorimetric (TBARS)
Ballesteros-Guzmán, A.K. 2019 [28]	Blood (Serum)	TAC [nmol/µL/µg protein]	Colorimetric
Rodriguez-Cano A. M. 2022 [19]	Blood (Serum and Plasma)	MDA [nmol/mg dry weight]	Colorimetric (Gérard-Monnier)
Rodriguez-Cano A. M. 2022 [19]		TAC [nmol/mg protein]	Colorimetric (CUPRAC)
Rodriguez-Cano A. M. 2022 [19]		PC [pmol trolox equivalent/mg protein]	DNPH method
Chen, X. 2003 [21]		GPx [mU/mg Hb]	Colorimetric
Morales, E. 2022 [13]	Urine * and Blood (Serum and Plasma)	Isoprostane [ng/mg creatinine]	ELISA
Morales, E. 2022 [13]		8OHdG [ng/mL]	ELISA
Scholl, T. O. 2005 [20]		Isoprostane [ng/mg creatinine]	ELISA
Scholl, T. O. 2005 [20]		TAC [µmol/L]	ELISA

* Urine samples are always spot urine. Abbreviations: OS: oxidative stress; MDA: malondialdehyde; HPLC: High-Performance Liquid Chromatography; 8OHdG: 8-hydroxy-2′-deoxyguanosine; ELISA: enzyme-linked immunosorbent assay; MS: Mass Spectrometry; CoQ10: Coenzyme Q10; TBARS: thiobarbituric acid reactive substances; NO: nitric oxide; TAC: total antioxidant capacity; CUPRAC: CUPric Reducing Antioxidant Capacity; PC: protein carbonylation; DNPH: dinitropenylhydrazine; GPx: glutathione peroxidase.

**Table 3:** Association between oxidative stress and dietary exposure.

Biomarker	Quantification Rate in Included Studies	Significant Association between OS and Diet in the Included Studies	Dietary Exposure
MDA	46%	83%	Dietary pattern/fruit and vegetables intake
Isoprostane	23%	67%	Antioxidant diet/fat and PUFA intake
8OHdG	31%	75%	Dietary pattern/fat and PUFA intake/vitamins intake
TAC	23%	33%	Dietary pattern
Others	31%	75%	Fat and PUFA intake/fruit and vegetables intake

Abbreviations: MDA: malondialdehyde; PUFA: polyunsaturated fatty acids; 8OHdG: 8-hydroxy-2′-deoxyguanosine; TAC: total antioxidant capacity.

## Data Availability

The data presented in this study are available in the original articles that were included in the systematic review.

## Appendix A

Search strategy used for each database.

PubMed

#1 “Oxidative Stress”[Mesh] OR “Antioxidants”[Mesh] OR oxida*[tiab] OR antioxida*[tiab]

#2 “Malondialdehyde”[Mesh] OR malondialdehyde[tiab] OR malonylaldehyde[tiab] OR malonaldehyde[tiab] OR malonyldialdehyde[tiab] OR MDA[tiab]

#3 “8-Hydroxy-2′-Deoxyguanosine”[Mesh] OR “8-hydroxy-2-deoxyguanosine”[tiab] OR “8-hydroxy-deoxyguanosine”[tiab] OR “8-hydroxydeoxyguanosine”[tiab] OR “8-hydroxyguanine”[tiab] OR “8-hydroxy-guanine”[tiab] OR “8-Oxo-2-Deoxyguanosine”[tiab] OR “8-Oxo-Deoxyguanosine”[tiab] OR “8-oxo-dGuo”[tiab] OR 8-Ohdg[tiab] OR 8OHdG[tiab] OR 8-OH-dG[tiab] OR 8-ohg[tiab] OR 8-hydroxy-g[tiab] OR 8-hydroxy-dg[tiab] OR 8-oxodG[tiab] OR 8-oxodGuo[tiab] OR 8-oxo-dG[tiab] OR 8-OH-2dG*[tiab] OR 8-Isoprostane*[tiab]

#4 “F2-Isoprostanes”[Mesh] OR Isoprostane[tiab] OR F2-Isoprostane*[tiab]

#5 “Dinoprost”[Mesh] OR dinoprost[tiab] OR 15-f2t-Isoprostane[tiab] OR 8-iso-PGF2a[tiab] OR 8-Isoprostanerostaglandin-f2[tiab] OR 8-iso-prostaglandin-f2[tiab] OR 8-iso-PGF2a[tiab] OR 8-epi-prostaglandin-F2alpha[tiab] OR 8-epi-prostaglandin-f2alpha[tiab] OR 8-epiprostaglandin-f2alpha[tiab] OR 8-epi-PGF2alpha[tiab]

#6 “Allantoin”[Mesh] OR allantoin*[tiab] OR dioxo-4-imidazolidinyl*[tiab] OR glyoxyldiureide*[tiab] OR 5-ureidohydantoin*[tiab]

#7 “total antioxidant capacity”[tiab] OR “total anti-oxidant capacity”[tiab] OR “total antioxidant power”[tiab] OR TAC[tiab] OR ToAC[tiab] OR “total anti-oxidant power”[tiab] OR TAP[tiab]

#8 “trolox equivalent antioxidant capacity”[tiab] OR “trolox equivalent anti-oxidant capacity”[tiab] OR “trolox equivalence antioxidant capacity”[tiab] OR “trolox equivalence anti-oxidant capacity”[tiab] OR TEAC[tiab]

#9 “Thiobarbituric Acid Reactive Substances”[Mesh] OR TBARS[tiab] OR “thiobarbituric acid reactive substance*”[tiab]

#10 “Glutathione”[Mesh] OR “Glutathione Peroxidase”[Mesh] OR glutathion*[tiab] OR GSH[tiab] OR GSSH[tiab] OR GSSG[tiab] OR GPX[tiab]

#11 “Uric Acid”[Mesh] OR “uric acid”[tiab] OR UA[tiab]

#12 “Superoxide Dismutase”[Mesh] OR dismutase*[tiab] OR SOD[tiab]

#13 “Lipid Peroxides”[Mesh] OR “lipid peroxid*”[tiab] OR hydroperoxid*[tiab] OR lipoperoxid*[tiab] OR hydro-peroxid*[tiab] OR lipo-peroxid*[tiab]

#14 “ferric reducing”[tiab] OR “ferric ion reducing”[tiab] OR FRAP[tiab]

#15 “oxygen radical absor*”[tiab] OR ORAC[tiab]

#16 “cupric reducing”[tiab] OR CUPRAC[tiab]

#17 “hydroxyl radical”[tiab] OR HORAC[tiab]

#18 “potassium ferricyanide reducing power”[tiab] OR PFRAP[tiab]

#19 “total peroxyl radical trapping”[tiab] OR “total reactive antioxidant potential”[tiab] OR “total reactive anti-oxidant potential”[tiab] OR “total radical-trapping antioxidant parameter”[tiab] OR “total radical-trapping anti-oxidant parameter”[tiab] OR TRAP[tiab]

#20 picrylhydrazyl[tiab] OR picryl-hydrazyl[tiab] OR DPPH[tiab]

#21 “total oxyradical scavenging capacity”[tiab] OR TOSC[tiab]

#22 #1 OR #2 OR #3 OR #4 OR #5 OR #6 OR #7 OR #8 OR #9 OR #10 OR #11 OR #12 OR #13 OR #14 OR #15 OR #16 OR #17 OR #18 OR #19 OR #20 OR #21

#23 “Prenatal Nutritional Physiological Phenomena”[Mesh]

#24 “Pregnancy”[Mesh] OR “Pregnant Women”[Mesh] OR “Pregnancy Outcome”[Mesh] OR pregnan*[tiab] OR gestation[tiab] OR gestations[tiab] OR childbearing[tiab] OR child-bearing[tiab]

#25 “Diet”[Mesh] OR “Food”[Mesh] OR “Eating”[Mesh] OR “Feeding Behavior”[Mesh] OR “Diet Therapy”[Mesh] OR “Maternal Nutritional Physiological Phenomena”[Mesh:noexp] OR diet[tiab] OR diets[tiab] OR dietary[tiab] OR dietetic*[tiab] OR dieting[tiab] OR dieto*[tiab] OR food[tiab] OR foods[tiab] OR eating[tiab] OR nutrit*[tiab] OR nutrie*[tiab] OR intake*[tiab] OR consumption*[tiab]

#26 #24 AND #25

#27 #23 OR #26

#28 “Observational Study”[Publication Type] OR “Epidemiologic Studies”[Mesh] OR observational[tiab] OR epidemiologic[tiab] OR epidemiological[tiab] OR case-control*[tiab] OR retrospective*[tiab] OR cohort*[tiab] OR longitudinal*[tiab] OR prospective*[tiab] OR cross-section*[tiab] OR transversal*[tiab] OR population-based[tiab] OR “time series”[tiab] OR “Regression Analysis”[Mesh] OR regression[tiab] OR “Matched-Pair Analysis”[Mesh] OR matching[tiab] OR matched[tiab] OR “Prevalence”[Mesh] OR prevalen*[tiab] OR “Incidence”[Mesh] OR incidence[tiab]

#29 #22 AND #27 AND #28

#30 “Animals”[Mesh] NOT “Humans”[Mesh]

#31 #29 NOT #30

Embase

#1 ‘oxidative stress’/exp OR ‘antioxidant’/exp OR oxida*:ti,ab,kw OR antioxida*:ti,ab,kw

#2 ‘malonaldehyde’/exp OR malondialdehyde:ti,ab,kw OR malonylaldehyde:ti,ab,kw OR malonaldehyde:ti,ab,kw OR malonyldialdehyde:ti,ab,kw OR MDA:ti,ab,kw

#3 ‘8 hydroxydeoxyguanosine’/exp OR ‘8-hydroxy-2-deoxyguanosine’:ti,ab,kw OR ‘8-hydroxy-deoxyguanosine’:ti,ab,kw OR ‘8-hydroxydeoxyguanosine’:ti,ab,kw OR ‘8-hydroxyguanine’:ti,ab,kw OR ‘8-hydroxy-guanine’:ti,ab,kw OR ‘8-Oxo-2-Deoxyguanosine’:ti,ab,kw OR ‘8-Oxo-Deoxyguanosine’:ti,ab,kw OR ‘8-oxo-dGuo’:ti,ab,kw OR 8-Ohdg:ti,ab,kw OR 8OHdG:ti,ab,kw OR 8-OH-dG:ti,ab,kw OR 8-ohg:ti,ab,kw OR 8-hydroxy-g:ti,ab,kw OR 8-hydroxy-dg:ti,ab,kw OR 8-oxodG:ti,ab,kw OR 8-oxodGuo:ti,ab,kw OR 8-oxo-dG:ti,ab,kw OR 8-OH-2dG*:ti,ab,kw OR 8-Isoprostane*:ti,ab,kw

#4 ‘Isoprostane derivative’/exp OR Isoprostane:ti,ab,kw OR F2-Isoprostane*:ti,ab,kw

#5 ‘prostaglandin F2 alpha’/exp OR dinoprost:ti,ab,kw OR 15-f2t-Isoprostane:ti,ab,kw OR 8-iso-PGF2a:ti,ab,kw OR 8-Isoprostanerostaglandin-f2:ti,ab,kw OR 8-iso-prostaglandin-f2:ti,ab,kw OR 8-iso-PGF2a:ti,ab,kw OR 8-epi-prostaglandin-F2alpha:ti,ab,kw OR 8-epi-prostaglandin-f2alpha:ti,ab,kw OR 8-epiprostaglandin-f2alpha:ti,ab,kw OR 8-epi-PGF2alpha:ti,ab,kw

#6 ‘allantoin’/exp OR allantoin*:ti,ab,kw OR dioxo-4-imidazolidinyl*:ti,ab,kw OR glyoxyldiureide*:ti,ab,kw OR 5-ureidohydantoin*:ti,ab,kw

#7 ‘total antioxidant capacity’/exp OR ‘total antioxidant capacity’:ti,ab,kw OR ‘total anti-oxidant capacity’:ti,ab,kw OR ‘total antioxidant power’:ti,ab,kw OR TAC:ti,ab,kw OR ToAC:ti,ab,kw OR ‘total anti-oxidant power’:ti,ab,kw OR TAP:ti,ab,kw

#8 ‘trolox equivalent antioxidant capacity’/exp OR ‘trolox equivalent antioxidant capacity’:ti,ab,kw OR ‘trolox equivalent anti-oxidant capacity’:ti,ab,kw OR ‘trolox equivalence antioxidant capacity’:ti,ab,kw OR ‘trolox equivalence anti-oxidant capacity’:ti,ab,kw OR TEAC:ti,ab,kw

#9 ‘thiobarbituric acid reactive substance’/exp OR TBARS:ti,ab,kw OR ‘thiobarbituric acid reactive substance*’:ti,ab,kw

#10 ‘glutathione’/exp OR ‘glutathione peroxidase’/exp OR glutathion*:ti,ab,kw OR GSH:ti,ab,kw OR GSSH:ti,ab,kw OR GSSG:ti,ab,kw OR GPX:ti,ab,kw

#11 ‘uric acid’/exp OR ‘uric acid’:ti,ab,kw OR UA:ti,ab,kw

#12 ‘superoxide dismutase’/exp OR dismutase*:ti,ab,kw OR SOD:ti,ab,kw

#13 ‘antioxidant assay’/exp OR ‘lipid peroxide’/exp OR ‘lipid peroxid*’:ti,ab,kw OR hydroperoxid*:ti,ab,kw OR lipoperoxid*:ti,ab,kw OR hydro-peroxid*:ti,ab,kw OR lipo-peroxid*:ti,ab,kw

#14 ‘ferric reducing antioxidant power’/exp OR ‘ferric reducing’:ti,ab,kw OR ‘ferric ion reducing’:ti,ab,kw OR FRAP:ti,ab,kw

#15 ‘oxygen radical absorbance capacity’/exp OR ‘oxygen radical absor*’:ti,ab,kw OR ORAC:ti,ab,kw

#16 ‘cupric reducing antioxidant capacity’/exp OR ‘cupric reducing’:ti,ab,kw OR CUPRAC:ti,ab,kw

#17 ‘hydroxyl radical’:ti,ab,kw OR HORAC:ti,ab,kw

#18 ‘potassium ferricyanide reducing power’:ti,ab,kw OR PFRAP:ti,ab,kw

#19 ‘total radical-trapping antioxidant parameter’/exp OR ‘total peroxyl radical trapping’:ti,ab,kw OR ‘total reactive antioxidant potential’:ti,ab,kw OR ‘total reactive anti-oxidant potential’:ti,ab,kw OR ‘total radical-trapping antioxidant parameter’:ti,ab,kw OR ‘total radical-trapping anti-oxidant parameter’:ti,ab,kw OR TRAP:ti,ab,kw

#20 ‘1,1 diphenyl 2 picrylhydrazyl’/exp OR picrylhydrazyl:ti,ab,kw OR picryl-hydrazyl:ti,ab,kw OR DPPH:ti,ab,kw

#21 ‘total oxyradical scavenging capacity’:ti,ab,kw OR TOSC:ti,ab,kw

#22 #1 OR #2 OR #3 OR #4 OR #5 OR #6 OR #7 OR #8 OR #9 OR #10 OR #11 OR #12 OR #13 OR #14 OR #15 OR #16 OR #17 OR #18 OR #19 OR #20 OR #21

#23 ‘pregnancy’/exp OR ‘pregnant woman’/exp OR ‘pregnancy outcome’/exp OR pregnan*:ti,ab,kw OR gestation:ti,ab,kw OR gestations:ti,ab,kw OR childbearing:ti,ab,kw OR child-bearing:ti,ab,kw

#24 ‘diet’/exp OR ‘dietary pattern’/exp OR ‘food’/exp OR ‘food intake’/exp OR ‘dietary intake’/exp OR ‘feeding behavior’/exp OR ‘diet therapy’/exp OR ‘maternal nutrition’/exp OR diet:ti,ab,kw OR diets:ti,ab,kw OR dietary:ti,ab,kw OR dietetic*:ti,ab,kw OR dieting:ti,ab,kw OR dieto*:ti,ab,kw OR food:ti,ab,kw OR foods:ti,ab,kw OR eating:ti,ab,kw OR nutrit*:ti,ab,kw OR nutrie*:ti,ab,kw OR intake*:ti,ab,kw OR consumption*:ti,ab,kw

#25 #22 AND #23 AND #24

#26 ‘observational study’/exp OR ‘case control study’/exp OR ‘retrospective study’/exp OR ‘cohort analysis’/exp OR ‘longitudinal study’/exp OR ‘prospective study’/exp OR ‘cross-sectional study’/exp OR observational:ti,ab,kw OR epidemiologic:ti,ab,kw OR epidemiological:ti,ab,kw OR case-control*:ti,ab,kw OR retrospective*:ti,ab,kw OR cohort*:ti,ab,kw OR longitudinal*:ti,ab,kw OR prospective*:ti,ab,kw OR cross-section*:ti,ab,kw OR transversal*:ti,ab,kw OR population-based:ti,ab,kw OR ‘time series’:ti,ab,kw OR ‘regression model’/exp OR regression:ti,ab,kw OR matching:ti,ab,kw OR matched:ti,ab,kw OR ‘prevalence’/exp OR prevalen*:ti,ab,kw OR ‘incidence’/exp OR incidence:ti,ab,kw

#27 #25 AND #26

#28 (‘animal’/de OR ‘animal experiment’/exp) NOT (‘human’/exp OR ‘human experiment’/exp)

#29 #27 NOT #28

#30 #29 NOT ‘conference abstract’/it

Food Science and Technology Abstracts (FSTA)

S1 DE “OXIDATIVE STRESS” OR DE “ANTIOXIDANTS” OR DE “NATURAL ANTIOXIDANTS” OR TI oxida* OR TI antioxida* OR AB oxida* OR AB antioxida*

S2 DE “MALONDIALDEHYDE” OR TI malondialdehyde OR TI malonylaldehyde OR TI malonaldehyde OR TI malonyldialdehyde OR TI “MDA” OR AB malondialdehyde OR AB malonylaldehyde OR AB malonaldehyde OR AB malonyldialdehyde OR AB “MDA”

S3 TI “8-hydroxy-2-deoxyguanosine” OR TI “8-hydroxy-deoxyguanosine” OR TI “8-hydroxydeoxyguanosine” OR TI “8-hydroxyguanine” OR TI “8-hydroxy-guanine” OR TI “8-Oxo-2-Deoxyguanosine” OR TI “8-Oxo-Deoxyguanosine” OR TI “8-oxo-dGuo” OR TI “8-Ohdg” OR TI 8OHdG OR TI “8-OH-dG” OR TI “8-ohg” OR TI “8-hydroxy-g” OR TI “8-hydroxy-dg” OR TI “8-oxodG” OR TI “8-oxodGuo” OR TI “8-oxo-dG” OR TI “8-OH-2dG*” OR TI “8-Isoprostane*” OR AB “8-hydroxy-2-deoxyguanosine” OR AB “8-hydroxy-deoxyguanosine” OR AB “8-hydroxydeoxyguanosine” OR AB “8-hydroxyguanine” OR AB “8-hydroxy-guanine” OR AB “8-Oxo-2-Deoxyguanosine” OR AB “8-Oxo-Deoxyguanosine” OR AB “8-oxo-dGuo” OR AB “8-Ohdg” OR AB 8OHdG OR AB “8-OH-dG” OR AB “8-ohg” OR AB “8-hydroxy-g” OR AB “8-hydroxy-dg” OR AB “8-oxodG” OR AB “8-oxodGuo” OR AB “8-oxo-dG” OR AB “8-OH-2dG*” OR AB “8-Isoprostane*”

S4 TI Isoprostane OR TI “F2-Isoprostane*” OR AB Isoprostane OR AB “F2-Isoprostane*”

S5 TI dinoprost OR TI “15-f2t-Isoprostane” OR TI “8-iso-PGF2a” OR TI “8-Isoprostanerostaglandin-f2” OR TI “8-iso-prostaglandin-f2” OR TI “8-iso-PGF2a” OR TI “8-epi-prostaglandin-F2alpha” OR TI “8-epi-prostaglandin-f2alpha” OR TI “8-epiprostaglandin-f2alpha” OR TI “8-epi-PGF2alpha” OR AB dinoprost OR AB “15-f2t-Isoprostane” OR AB “8-iso-PGF2a” OR AB “8-Isoprostanerostaglandin-f2” OR AB “8-iso-prostaglandin-f2” OR AB “8-iso-PGF2a” OR AB “8-epi-prostaglandin-F2alpha” OR AB “8-epi-prostaglandin-f2alpha” OR AB “8-epiprostaglandin-f2alpha” OR AB “8-epi-PGF2alpha”

S6 DE “ALLANTOIN” OR TI allantoin* OR TI “dioxo-4-imidazolidinyl*” OR TI glyoxyldiureide* OR TI “5-ureidohydantoin*” OR AB allantoin* OR AB “dioxo-4-imidazolidinyl*” OR AB glyoxyldiureide* OR AB “5-ureidohydantoin*”

S7 TI “total antioxidant capacity” OR TI “total anti-oxidant capacity” OR TI “total antioxidant power” OR TI “TAC” OR TI “ToAC” OR TI “total anti-oxidant power” OR TI “TAP” OR AB “total antioxidant capacity” OR AB “total anti-oxidant capacity” OR AB “total antioxidant power” OR AB “TAC” OR AB “ToAC” OR AB “total anti-oxidant power” OR AB “TAP”

S8 TI “trolox equivalent antioxidant capacity” OR TI “trolox equivalent anti-oxidant capacity” OR TI “trolox equivalence antioxidant capacity” OR TI “trolox equivalence anti-oxidant capacity” OR TI “TEAC” OR AB “trolox equivalent antioxidant capacity” OR AB “trolox equivalent anti-oxidant capacity” OR AB “trolox equivalence antioxidant capacity” OR AB “trolox equivalence anti-oxidant capacity” OR AB “TEAC”

S9 DE “TBARS” OR TI “TBARS” OR TI “thiobarbituric acid reactive substance*” OR AB “TBARS” OR AB “thiobarbituric acid reactive substance*”

S10 DE “GLUTATHIONE” OR DE “GLUTATHIONE PEROXIDASES” OR TI glutathion* OR TI “GSH” OR TI “GSSH” OR TI “GSSG” OR TI “GPX” OR AB glutathion* OR AB “GSH” OR AB “GSSH” OR AB “GSSG” OR AB “GPX”

S11 DE “URIC ACID” OR TI “uric acid” OR TI “UA” OR AB “uric acid” OR AB “UA”

S12 DE “SUPEROXIDE DISMUTASES” OR TI dismutase* OR TI “SOD” OR AB dismutase* OR AB “SOD”

S13 DE “HYDROPEROXIDES” OR TI “lipid peroxid*” OR TI hydroperoxid* OR TI lipoperoxid* OR TI “hydro-peroxid*” OR TI “lipo-peroxid*” OR AB “lipid peroxid*” OR AB hydroperoxid* OR AB lipoperoxid* OR AB “hydro-peroxid*” OR AB “lipo-peroxid*”

S14 TI “ferric reducing” OR TI “ferric ion reducing” OR TI “FRAP” OR AB “ferric reducing” OR AB “ferric ion reducing” OR AB “FRAP”

S15 TI “oxygen radical absor*” OR TI “ORAC” OR AB “oxygen radical absor*” OR AB “ORAC”

S16 TI “cupric reducing” OR TI “CUPRAC” OR AB “cupric reducing” OR AB “CUPRAC”

S17 TI “hydroxyl radical” OR TI “HORAC” OR AB “hydroxyl radical” OR AB “HORAC”

S18 TI “potassium ferricyanide reducing power” OR TI “PFRAP” OR AB “potassium ferricyanide reducing power” OR AB “PFRAP”

S19 TI “total peroxyl radical trapping” OR TI “total reactive antioxidant potential” OR TI “total reactive anti-oxidant potential” OR TI “total radical-trapping antioxidant parameter” OR TI “total radical-trapping anti-oxidant parameter” OR TI “TRAP” OR AB “total peroxyl radical trapping” OR AB “total reactive antioxidant potential” OR AB “total reactive anti-oxidant potential” OR AB “total radical-trapping antioxidant parameter” OR AB “total radical-trapping anti-oxidant parameter” OR AB “TRAP”

S20 TI picrylhydrazyl OR TI “picryl-hydrazyl” OR TI “DPPH” OR AB picrylhydrazyl OR AB “picryl-hydrazyl” OR AB “DPPH”

S21 TI “total oxyradical scavenging capacity” OR TI “TOSC” OR AB “total oxyradical scavenging capacity” OR AB “TOSC”

S22 S1 OR S2 OR S3 OR S4 OR S5 OR S6 OR S7 OR S8 OR S9 OR S10 OR S11 OR S12 OR S13 OR S14 OR S15 OR S16 OR S17 OR S18 OR S19 OR S20 OR S21

S23 DE “PRENATAL NUTRITION”

S24 DE “PREGNANCY” OR TI pregnan* OR TI gestation OR TI gestations OR TI childbearing OR TI “child-bearing” OR AB pregnan* OR AB gestation OR AB gestations OR AB childbearing OR AB “child-bearing”

S25 DE “DIET” OR DE “DIET QUALITY” OR DE “DIET THERAPY” OR DE “DIETARY PATTERNS” OR DE “FATS HIGH DIET” OR DE “FLEXITARIAN DIET” OR DE “HEALTHY DIETS” OR DE “INTAKE” OR DE “MEDITERRANEAN DIET” OR DE “PROTEINS HIGH DIET” OR DE “PROTEINS LOW DIET” OR DE “PERSONALIZED DIET” OR DE “PLANT-BASED DIETS” OR DE “SUSTAINABLE DIETS” OR DE “VEGETARIAN DIET” OR DE “WESTERN DIET” OR DE “DIET THERAPY” OR DE “CALORIES LOW DIET” OR DE “CARBOHYDRATES LOW DIET” OR DE “DASH DIET” OR DE “DIETARY RESTRICTION” OR DE “FATS LOW DIET” OR DE “GLUTEN LOW DIET” OR DE “KETOGENIC DIET” OR DE “LOW FODMAP DIET” OR DE “SODIUM LOW DIET” OR DE “WT. LOSS DIET” OR DE “FOODS” OR DE “ANIMAL FOODS” OR DE “ALTERNATIVES” OR DE “CALORIES HIGH FOODS” OR DE “CALORIES LOW FOODS” OR DE “CARBOHYDRATES LOW FOODS” OR DE “CHOLESTEROL LOW FOODS” OR DE “CONDIMENTS” OR DE “FATS LOW FOODS” OR DE “FREE RANGE FOODS” OR DE “FRESH PRODUCE” OR DE “FATS HIGH FOODS” OR DE “FOOD MATRIX” OR DE “HEALTHY FOODS” OR DE “LACTOSE LOW FOODS” OR DE “LIQUID FOODS” OR DE “LONG LIFE FOODS” OR DE “MICROWAVEABLE FOODS” OR DE “MODEL FOODS” OR DE “NATURAL FOODS” OR DE “NOVEL FOODS” OR DE “ORGANIC FOODS” OR DE “PERISHABLE FOODS” OR DE “PLANT FOODS” OR DE “PROCESSED FOODS” OR DE “PROTEINS HIGH FOODS” OR DE “PROTEINS LOW FOODS” OR DE “REGIONAL FOODS” OR DE “SODIUM LOW FOODS” OR DE “SUGAR LOW FOODS” OR DE “UPCYCLED FOODS” OR DE “WILD FOODS” OR DE “EATING HABITS” OR DE “DISORDERED EATING” OR DE “FASTING” OR DE “FOOD PORTIONS” OR DE “HEALTHY EATING” OR DE “INTUITIVE EATING” OR DE “SNACKING” OR DE “MATERNAL NUTRITION”OR TI diet OR TI diets OR TI dietary OR TI dietetic* OR TI dieting OR TI dieto* OR TI food OR TI foods OR TI eating OR TI nutrit* OR TI nutrie* OR TI intake* OR TI consumption* OR AB diet OR AB diets OR AB dietary OR AB dietetic* OR AB dieting OR AB dieto* OR AB food OR AB foods OR AB eating OR AB nutrit* OR AB nutrie* OR AB intake* OR AB consumption*

S26 S24 AND S25

S27 S23 OR S26

S28 DE “CASE-CONTROL STUDIES” OR DE “COHORT STUDIES” OR TI observational OR TI epidemiologic OR TI epidemiological OR TI “case-control*” OR TI retrospective* OR TI cohort* OR TI longitudinal* OR TI prospective* OR TI “cross-section*” OR TI transversal* OR TI “population-based” OR TI “time series” OR TI regression OR TI matching OR TI matched OR TI prevalen* OR TI incidence OR AB observational OR AB epidemiologic OR AB epidemiological OR AB “case-control*” OR AB retrospective* OR AB cohort* OR AB longitudinal* OR AB prospective* OR AB “cross-section*” OR AB transversal* OR AB “population-based” OR AB “time series” OR AB regression OR AB matching OR AB matched OR AB prevalen* OR AB incidence

S29 S22 AND S27 AND S28

Web of Science

#1 TS = (oxida* OR antioxida* OR malondialdehyde OR malonylaldehyde OR malonaldehyde OR malonyldialdehyde OR MDA OR “8-hydroxy-2-deoxyguanosine” OR “8-hydroxy-deoxyguanosine” OR “8-hydroxydeoxyguanosine” OR “8-hydroxyguanine” OR “8-hydroxy-guanine” OR “8-Oxo-2-Deoxyguanosine” OR “8-Oxo-Deoxyguanosine” OR “8-oxo-dGuo” OR 8-Ohdg OR 8OHdG OR 8-OH-dG OR 8-ohg OR 8-hydroxy-g OR 8-hydroxy-dg OR 8-oxodG OR 8-oxodGuo OR 8-oxo-dG OR 8-OH-2dG* OR 8-Isoprostane* OR Isoprostane OR F2-Isoprostane* OR dinoprost OR 15-f2t-Isoprostane OR 8-iso-PGF2a OR 8-Isoprostanerostaglandin-f2 OR 8-iso-prostaglandin-f2 OR 8-iso-PGF2a OR 8-epi-prostaglandin-F2alpha OR 8-epi-prostaglandin-f2alpha OR 8-epiprostaglandin-f2alpha OR 8-epi-PGF2alpha OR allantoin* OR dioxo-4-imidazolidinyl* OR glyoxyldiureide* OR 5-ureidohydantoin* OR “total antioxidant capacity” OR “total anti-oxidant capacity” OR “total antioxidant power” OR TAC OR ToAC OR “total anti-oxidant power” OR TAP OR “trolox equivalent antioxidant capacity” OR “trolox equivalent anti-oxidant capacity” OR “trolox equivalence antioxidant capacity” OR “trolox equivalence anti-oxidant capacity” OR TEAC OR TBARS OR “thiobarbituric acid reactive substance*” OR glutathion* OR GSH OR GSSH OR GSSG OR GPX OR “uric acid” OR UA OR dismutase* OR SOD OR “lipid peroxid*” OR hydroperoxid* OR lipoperoxid* OR hydro-peroxid* OR lipo-peroxid* OR “ferric reducing” OR “ferric ion reducing” OR FRAP OR “oxygen radical absor*” OR ORAC OR “cupric reducing” OR CUPRAC OR “hydroxyl radical” OR HORAC OR “potassium ferricyanide reducing power” OR PFRAP OR “total peroxyl radical trapping” OR “total reactive antioxidant potential” OR “total reactive anti-oxidant potential” OR “total radical-trapping antioxidant parameter” OR “total radical-trapping anti-oxidant parameter” OR TRAP OR picrylhydrazyl OR picryl-hydrazyl OR DPPH OR “total oxyradical scavenging capacity” OR TOSC)

#2 TS = (pregnan* OR gestation OR gestations OR childbearing OR child-bearing)

#3 TS = (diet OR diets OR dietary OR dietetic* OR dieting OR dieto* OR food OR foods OR eating OR nutrit* OR nutrie* OR intake* OR consumption*)

#4 #1 AND #2 AND #3

#5 TS = (observational OR epidemiologic OR epidemiological OR case-control* OR retrospective* OR cohort* OR longitudinal* OR prospective* OR cross-section* OR transversal* OR population-based OR “time series” OR regression OR matching OR matched OR prevalen* OR incidence)

#6 #4 AND #5
